# Evaluating the impact of visceral fat on the outcomes of frozen embryo transfer via bioelectrical impedance analysis

**DOI:** 10.3389/fendo.2024.1474201

**Published:** 2025-01-10

**Authors:** Danyu Ni, Yi Wei, Qijun Xie, Xinyu Wang, Kaidi Yu, Wei Jiang, Ye Yang, Xiufeng Ling

**Affiliations:** Department of Reproductive Medicine, Women’s Hospital of Nanjing Medical University, Nanjing Women and Children’s Healthcare Hospital, Nanjing, China

**Keywords:** visceral fat area (VFA), bioelectrical impedance analysis (BIA), frozen embryo transfer (FET), clinical pregnancy rate (CPR), live birth rate (LBR)

## Abstract

**Objectives:**

The increasing prevalence of obesity underscores the need to explore its impact on assisted reproductive technology (ART) outcomes. This study aims to evaluate the association between visceral fat area (VFA), measured by bioelectrical impedance analysis (BIA), and pregnancy outcomes following frozen embryo transfer (FET).

**Methods:**

In this retrospective clinical study, the data of 1,510 patients who underwent FET between April 2022 and April 2023 were analyzed. The VFA was measured by BIA, and patients were categorized into low and high VFA groups based on a threshold of 65 cm². Pregnancy outcomes were compared between the two groups. Univariable and multivariate logistic regression analyses, along with restricted cubic spline (RCS) modeling, were used to adjust for age, body mass index (BMI), and basal estradiol (E2) levels to determine the relationship between VFA and FET outcomes.

**Results:**

There were significant differences in baseline characteristics and outcomes between the two groups. The high VFA group was characterized by older age and a lower basal estradiol (E2) level. The biochemical pregnancy rate, implantation rate, clinical pregnancy rate (CPR), and live birth rate (LBR) were significantly lower in the high VFA group. Logistic regression revealed a significant negative correlation between the high VFA group and both CPR and LBR. The RCS model demonstrated that the VFA was nonlinearly correlated with CPR and LBR. Subgroup analysis showed that among individuals under 35 years of age or with a BMI < 24, high VFA was significantly associated with poorer CPR and LBR.

**Conclusions:**

High VFA is associated with poorer pregnancy outcomes after FET in female patients with infertility, with both CPR and LBR decreasing as VFA increases. Clinicians should consider VFA as an important reference for targeted fat management interventions to optimize reproductive success, especially when VFA exceeds 65 cm².

## 1 Introduction

The increasing prevalence of obesity is a significant public health challenge that extends beyond metabolic disorders to encompass reproductive health (1, 2). Evidence suggests that obesity adversely affects both male and female reproductive health, leading to complications ranging from hormonal imbalances to reduced efficacy of fertility treatments (3). In patients undergoing assisted reproductive technology (ART), obesity is associated with diminished ovarian response to stimulation, which negatively impacts oocyte quality and endometrial function. This, in turn, increases miscarriage rates and reduces both the clinical pregnancy rate (CPR) (odds ratio [*OR*] 0.50, 95% confidence interval [*CI*] 0.31–0.82) and the live birth rate (LBR) (*OR* 0.51, 95% *CI* 0.29–0.87) (4). Therefore, identifying an indicator that accurately reflects the impact of obesity on ART outcomes and using this indicator as a basis to provide targeted adiposity interventions is critical to improving the success of ART.

While body mass index (BMI) has traditionally been used as the standard measure of obesity, it fails to capture the complexities of body composition, particularly the impact of fat distribution on fertility and pregnancy outcomes (5–7). This limitation can obscure how fat, particularly visceral adiposity, influences the endocrine environment and reproductive function. In light of these shortcomings, the present study shifts focus from BMI to a more precise measure of adiposity that directly affects reproductive outcomes: visceral fat area (VFA). Visceral fat, which is stored within the abdominal cavity and around internal organs, has been associated with insulin resistance, inflammation, and altered sex hormone metabolism—factors known to negatively impact fertility and pregnancy outcomes (8–10). Among the methods available for measuring visceral fat, such as computed tomography, magnetic resonance imaging, ultrasound, and bioelectrical impedance analysis (BIA) (11), BIA was selected for the present study owing to its non-invasive nature, cost-effectiveness, convenience, and non-exposure to radiation, offering a practical approach for large-scale studies and clinical applications (12–14).

By using BIA to measure VFA, this study aims to evaluate the relationship between visceral fat and female reproductive outcomes. Isolating the effects of visceral fat will enable a more accurate assessment of its role in fertility and ART success, specifically frozen embryo transfer (FET). This nuanced understanding is essential for developing targeted interventions that could improve reproductive outcomes in women with elevated adiposity. By using VFA as a more precise measure of relevant fat content, we aim to elucidate the pathways through which adiposity affects fertility and pregnancy success, beyond the generalized and somewhat crude metric of BMI.

## 2 Materials and methods

### 2.1 Study population

This retrospective cohort study included female patients who underwent FET at the Reproductive Center of Women’s Hospital of Nanjing Medical University between April 2022 and April 2023. The exclusion criteria were as follows: severe chronic diseases (e.g., heart disease, diabetes mellitus, kidney disease), chromosomal abnormalities or genetic diseases in either partner, uterine malformations, endometrial thickness < 6 mm on the embryo transfer (ET) day, and incomplete cycle data.

### 2.2 Study groups and propensity score matching

Patients who met the inclusion criteria were stratified using a median VFA of 65 cm² as the cutoff point, dividing the patients into two groups: those with a VFA less than 65 cm² were defined as the low VFA group, and those with a VFA of 65 cm² or greater were categorized into the high VFA group. Propensity score matching (PSM) was employed to reduce potential bias between the groups. Patients were matched in a 1:1 ratio based on age, endometrial preparation protocols, and the type of embryos transferred (**Supplementary Table S1**).

### 2.3 Ethics approval and consent to participate

This retrospective cohort study was conducted according to the Declaration of Helsinki and was approved by the Ethics Committee of Women’s Hospital of Nanjing Medical University (approval number 2022KY-046). Given the retrospective and anonymized nature of the data analysis, the ethics committee waived the requirement for written informed consent.

### 2.4 Treatment protocol

#### 2.4.1 Body composition data collection before FET

On the initial day of progesterone (P) exposure, each patient’s body composition was measured by trained professionals using InBody 720, which utilizes BIA to rapidly evaluate body composition without the use of radiation (15). The patients were required to empty their bladder; remove their coats, shoes, socks, accessories, and any metallic items; and stand bare foot on the device, holding onto the measurement handles with the arms extended so that the arms and legs were in direct contact with the electrodes. Data were collected via a computer connected to the device.

#### 2.4.2 FET protocol and luteal phase support

In this study, the endometrial preparation protocols for patients undergoing FET included natural cycle (NC), ovulation induction (OI), and hormone replacement therapy (HRT). The choice of protocol was made by clinicians based on each patient’s medical history and professional judgment.

For evaluation of Day 3 (D3) cleavage-stage embryos, we applied the scoring system established by Scott et al. (16), defining embryos with a score of ≥3 as high-quality. For Day 5 (D5) or Day 6 (D6) blastocysts, grading was performed according to the criteria proposed by Gardner et al. (17), with blastocysts graded ≥3BB considered high-quality. All embryos underwent vitrification followed by thawing, and no embryos were subjected to preimplantation genetic testing (PGT).

Embryo transfer was performed under transabdominal ultrasound guidance. D3 embryos were transferred on Day 3 after progesterone administration (P+3), while D5 or D6 blastocysts were transferred on Day 5 after progesterone administration (P+5). The luteal phase was supported until the 10th week of pregnancy.

#### 2.4.3 Pregnancy outcome

Serum human chorionic gonadotropin (hCG) concentration was measured 14 days after FET to diagnose biochemical pregnancy. An ultrasound examination was conducted 28 days after FET to confirm clinical pregnancy based on the identification of a gestational sac. Miscarriage was defined as the natural termination of a clinical pregnancy before 28 weeks of gestation, with early miscarriage specifically referring to fetal loss before the 12th week of pregnancy. Live birth was defined as the delivery of a living infant at or beyond 28 weeks of gestation. The implantation rate was calculated as the ratio of gestational sacs to the number of embryos transferred. The primary outcome of interest was the CPR, while the secondary outcomes included the implantation rate, biochemical pregnancy rate, miscarriage rate and LBR.

### 2.5 Statistical analysis

All continuous variables are presented as the mean ± standard deviation, unless otherwise indicated. The comparison of descriptive statistics between the two groups was conducted using the independent-samples *t*-test. Categorical data were analyzed using Pearson’s chi-square (χ^2^) test or Fisher’s exact test, and the data are described as frequency (percentage). The Mann–Whitney U test was used to compare non-parametric variables. The univariable and multivariate logistic regression analysis was used to assess the correlation between the VFA and FET outcomes, with VFA categorized into high and low VFA groups based on a threshold of 65 cm². Restricted cubic splines (RCS) were applied to characterize the relationship between the VFA as a continuous variable and FET outcomes. Both the logistic regression analysis and RCS were adjusted for confounding variables, including age, BMI and basal E2 to minimize residual confounding. The statistical analyses were performed using SPSS software (v26.0, IBM Corp., Armonk, NY, US) and R statistical software (v4.2.0, R Foundation, Vienna, Austria). Statistical significance was defined as a two-sided *P*-value of <0.05.

## 3 Results

### 3.1 Baseline characteristics of study participants

**Figure 1:**
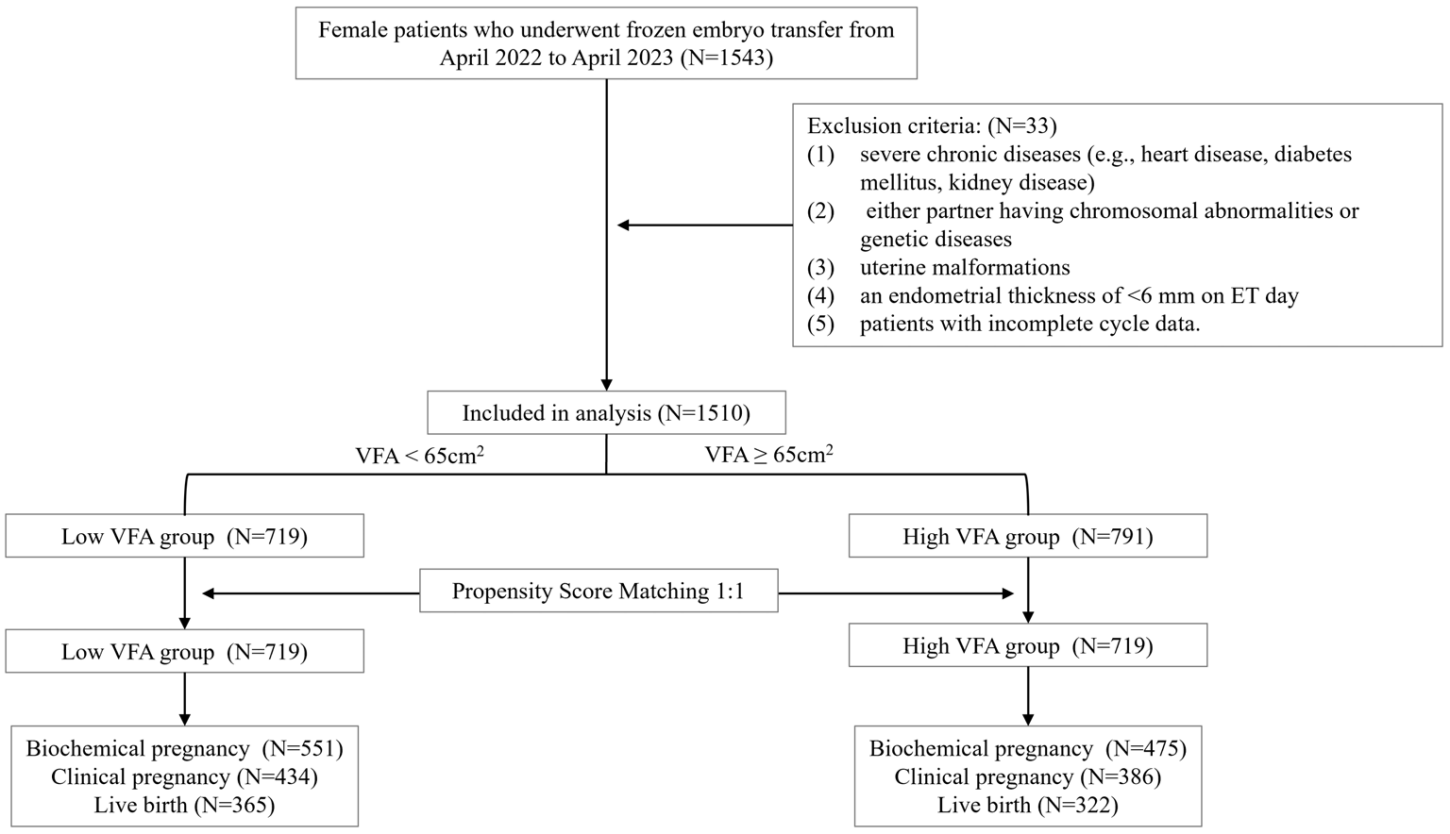
Flow chart of the participants included in this study. VFA, visceral fat area; ET, embryo transfer.

The study included 1,510 patients who underwent *in vitro* fertilization and embryo transfer cycles. The patients were categorized into two groups based on their VFA based on a cutoff threshold of 65 cm^2^: the low VFA group (VFA < 65 cm^2^, n = 719) and the high VFA group (VFA ≥ 65 cm^2^, n = 791). After PSM, 719 paired patients were included for analysis (**Figure 1**). The baseline characteristics are presented in **Table 1**. In this study, the low VFA group included 31 patients (4.3%) with a BMI that reached or exceeded 24 kg/m²; conversely, the high VFA group comprised 246 patients (34.2%) with a BMI that was below 24 kg/m². This suggests that even when the BMI is within the normal range or is low, individuals may still have a high accumulation of visceral fat, which could have adverse effects on reproductive health. Compared with the low VFA group, the high VFA group was significantly older (32.39 ± 4.25 vs. 31.31 ± 3.96 years, *P* < 0.001), had a significantly lower basal E2 levels (40.11 ± 16.76 vs. 42.98 ± 18.39 pg/mL, *P* = 0.002). The basal P, basal AMH level, type of infertility, factor of infertility, whether it was the first ET cycle, endometrial preparation protocols, endometrial thickness on ET day, type of embryos transferred, number of embryos transferred, number of high-quality embryos transferred were not significantly different between the two groups (all *P* **>** 0.05).

**Table 1:** Baseline characteristics of patients grouped by VFA.

Characteristics	Before Matching			After Matching		
	Low VFA Group	High VFA Group	*P* value	Low VFA Group	High VFA Group	*P* value
No. of patients	719	791		719	719	
Age, years	31.31±3.96	33.28±5.00	<0.001	31.31 ± 3.96	32.39 ± 4.25	<0.001
BMI group			<0.001			<0.001
< 24 kg/m^2^	688/719 (95.7%)	288 /791(36.4%)		688/719 (95.7%)	246/719 (34.2%)	
≥24 kg/m^2^	31/719 (4.3%)	503/791 (63.6%)		31/719 (4.3%)	473/719 (65.8%)	
Basal E2, pg/mL	42.98±18.39	40.11±16.74	0.011	42.98 ± 18.39	40.11 ± 16.76	0.002
Basal P, ng/mL	0.62±0.73	0.58±0.92	0.923	0.62 ± 0.73	0.60 ± 0.96	0.547
Basal AMH, ng/mL	5.10±3.96	4.87±3.80	0.682	5.10 ± 3.96	4.93 ± 3.81	0.429
Type of infertility, n (%)			0.083			0.596
Primary	330/719 (45.9%)	328/791(41.5%)		330/719 (45.9%)	320/719 (44.5%)	
Secondary	389/719 (54.1%)	463/791(58.5%)		389/719 (54.1%)	399/719 (55.5%)	
Factor of infertility, n (%)			0.025			0.092
PCOS	53/719(7.4%)	70/791(8.9%)		53/719 (7.4%)	70/719 (9.7%)	
Tuber factor	499/719(69.4%)	591/791(74.7%)		499/719 (69.4%)	521/719 (72.5%)	
Endometriosis	27/719(3.8%)	22/791(2.8%)		27/719 (3.8%)	22/719 (3.0%)	
Uterine factor	11/719(1.5%)	12/791(1.5%)		11/719 (1.5%)	12/719 (1.7%)	
Male factor	80/719(11.1%)	65/791(8.2%)		80/719 (11.1%)	63/719 (8.8%)	
Other	49/719(6.8%)	31/791(3.9%)		49/719 (6.8%)	31/719 (4.3%)	
First ET cycle, n (%)	564/719(78.4%)	621/791(78.5%)	0.975	564/719 (78.4%)	572/719 (79.6%)	0.605
Endometrial preparation protocols, n (%)			0.012			0.069
NC	187/719 (26.0%)	158/791(20.0%)		187/719 (26.0%)	153/719 (21.3%)	
OI	50/719 (7.00%)	71/791(9.0%)		50/719 (7.00%)	63/719 (8.8%)	
HRT	482/719 (67.0%)	562/791(71.0%)		482/719 (67.0%)	503/719 (69.9%)	
Endometrial thickness on ET day, mm	9.25±1.78	9.26±1.85	0.204	9.25 ± 1.78	9.29 ± 1.82	0.667
Type of embryos transferred, n (%)			0.024			0.273
D3	144/719(20.0%)	197/791(24.9%)		144/719 (20.0%)	161/719 (22.4%)	
D5/D6	575/719(80.0%)	594/791(75.1%)		575/719 (80.0%)	558/719 (77.6%)	
Number of embryos transferred, n (%)			0.650			0.670
1	311/719(43.3%)	333/791(42.1%)		311/719 (43.3%)	303/719 (42.1%)	
2	408/719(56.7%)	458/791(57.9%)		408/719 (56.7%)	416/719 (57.9%)	
Number of high-quality embryos transferred, n (%)			0.984			0.974
0	104/719 (14.5%)	117/791(14.8%)		104/719 (14.5%)	107/719 (14.9%)	
1	381/719 (53.0%)	418/791(52.8%)		381/719 (53.0%)	380/719 (52.8%)	
2	234/719 (32.5%)	256/791(32.4%)		234/719 (32.5%)	232/719 (32.3%)	

Data are presented as mean ± SD for continuous variables and n (%) for categorical variables. All *P* values were assessed with the use of student's t-test or χ^2^. VFA, visceral fat area; BMI, body mass index; E2, estradiol; P, progesterone; AMH, anti-müllerian hormone; PCOS, polycystic ovary syndrome; ET, embryo transfer; NC, natural cycle; OI, ovulation induction; HRT, hormone replacement therapy.

### 3.2 Clinical outcomes between two groups

We compared the clinical outcomes between the two groups (**Table 2**). Compared with the low VFA group, the high VFA group demonstrated a significantly lower biochemical pregnancy rate (66.1% vs. 76.6%, *P* < 0.001), implantation rate (42.3% vs. 47.1%, *P* = 0.005), CPR (53.7% vs. 60.4%, *P* = 0.011), and LBR (44.8% vs. 50.8%, *P* = 0.023). There was no significant difference in the miscarriage rate was observed between the two groups of patients (16.6% vs. 15.9%, *P* = 0.792).

**Table 2:** Clinical outcomes of patients grouped by VFA.

Outcomes	Low VFA Group	High VFA Group	*P* value
No. of patients	719	719	
Biochemical pregnancy rate, n (%)	551/719 (76.6%)	475/719 (66.1%)	<0.001
Implantation rate, n (%)	531/1127 (47.1%)	469/1136 (42.3%)	0.005
Clinical pregnancy rate, n (%)	434/719(60.4%)	386/719 (53.7%)	0.011
Miscarriage rate, n (%)	69/434 (15.9%)	64/386 (16.6%)	0.792
Live birth rate, n (%)	365/719 (50.8%)	322/719 (44.8%)	0.023

Data are presented as mean ± SD for continuous variables and n (%) for categorical variables. All *P* values were assessed with the use of student's t-test or χ^2^. VFA, Visceral fat area.

### 3.3 Relationship between VFA and clinical outcomes

**Table 3** displays the univariate and multiple logistic regression analysis that was utilized to explore the relationship between the VFA and clinical outcomes. In the univariable model, compared to the low VFA group, the biochemical pregnancy rate (*OR* 0.59, 95% *CI* 0.47 - 0.75, *P* < 0.001), CPR (*OR* 0.76, 95% *CI* 0.62 - 0.94, *P* = 0.011), and LBR (*OR* 0.79, 95% *CI* 0.64 - 0.97, *P* = 0.023) were significantly reduced in the high VFA group, while the miscarriage rate did not show a significant difference. In the multivariate model, after adjusting for age, BMI, and basal E2 levels, the CPR (*OR* 0.68, 95% *CI* 0.50 - 0.92, *P* = 0.013) and LBR (*OR* 0.73, 95% *CI* 0.54 - 0.98, *P =* 0.037) were significantly reduced in the high VFA group compared to the low VFA group, while the biochemical pregnancy rate (*OR* 0.78, 95% *CI* 0.56 - 1.08, *P =* 0.133) and miscarriage rate (*OR* 1.01, 95% *CI* 0.60 - 1.72, *P =* 0.959) showed no significant differences.

**Table 3:** Univariate and multiple logistic regression analysis of clinical outcomes.

Outcomes	Univariable		Multivariable	
	*OR* (95%CI)	*P* value	*OR* (95%CI)	*P* value
Biochemical pregnancy rate	0.59 (0.47 - 0.75)	<0.001	0.78 (0.56 - 1.08)	0.133
Clinical pregnancy rate	0.76 (0.62 - 0.94)	0.011	0.68 (0.50 - 0.92)	0.013
Miscarriage rate	1.05 (0.73 - 1.52)	0.792	1.01 (0.60 - 1.72)	0.959
Live birth rate	0.79 (0.64 - 0.97)	0.023	0.73 (0.54 - 0.98)	0.037

Low VFA group as reference. OR, odds ratios. Univariable model adjusts for: None. Multivariable model adjusts for: age, BMI, basal E2.

**Figure 2:**
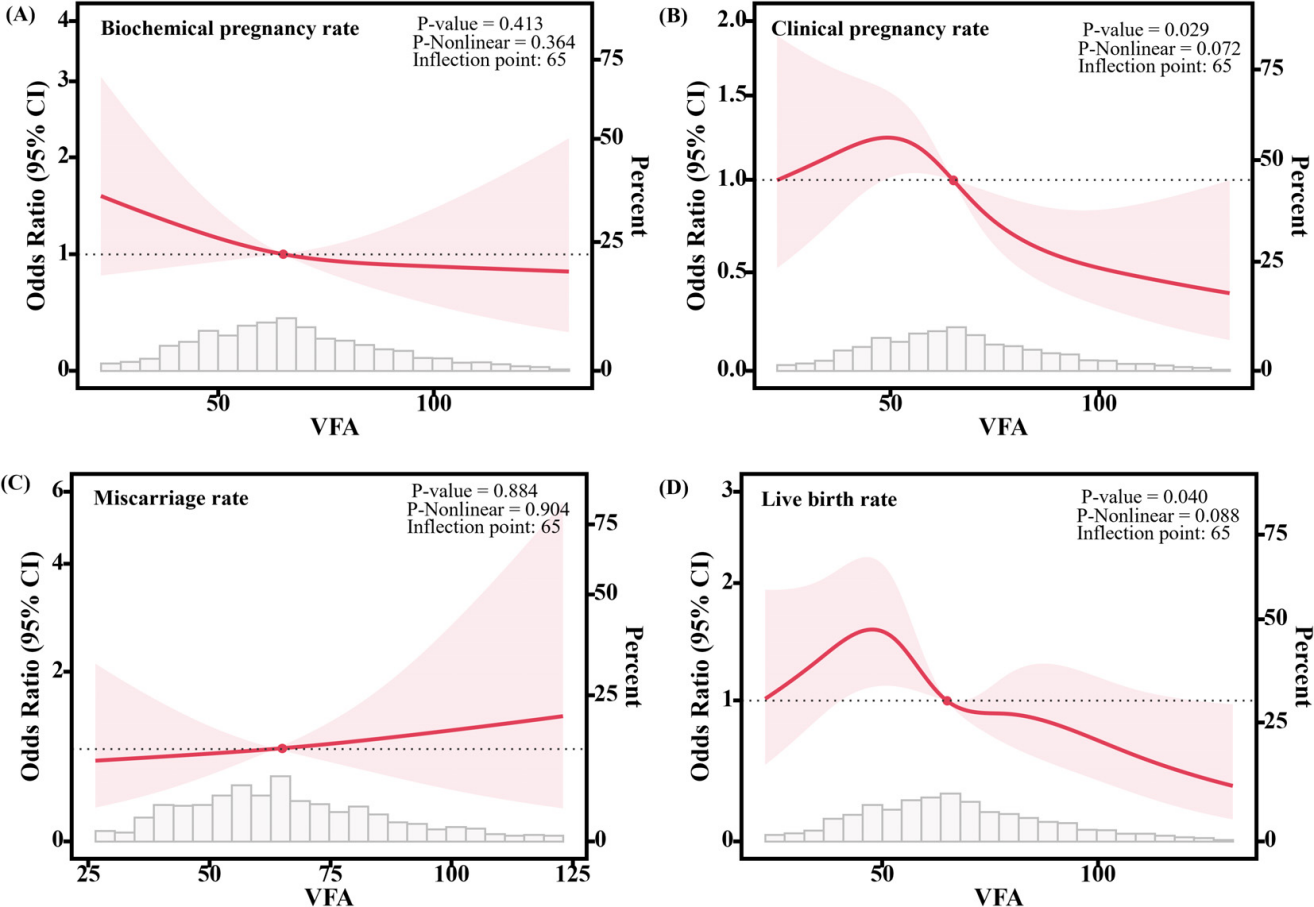
Association between VFA and clinical outcomes of frozen embryo transfer patients. **(A)** Relationship with the biochemical pregnancy rate. **(B)** Relationship with the clinical pregnancy rate. **(C)** Relationship with the miscarriage rate. **(D)** Relationship with the live birth rate. Solid lines show the estimation of the difference in clinical outcomes when using VFA=65 as the odds ratios. 95% confidence intervals (CIs) are indicated by shaded areas. The models were adjusted for age, BMI, basal E2. VFA, Visceral fat area.

**Figure 2** visualizes the association between the continuous variable of VFA and clinical outcomes after controlling for potential confounders such as age, BMI, and basal E2 levels using RCS models. We found that as VFA increased, the CPR (*P*-nonlinear = 0.072, *P* = 0.029) and LBR (*P*-nonlinear = 0.088, *P* = 0.040) significantly decreased, while the biochemical pregnancy rate (*P*-nonlinear = 0.364, *P* = 0.413) and miscarriage rate (*P*-nonlinear = 0.904, *P* = 0.884) were not significantly associated with VFA.

### 3.4 Subgroup analysis

As shown in **Table 4**, we performed subgroup analysis to stratify the association between high VFA group and clinical outcomes. In patients younger than 35 years, after adjusting for confounding factors, a high VFA was associated with a significant reduction in the CPR (*OR* 0.65, 95% *CI* 0.46 - 0.93, *P =* 0.018) and LBR (*OR* 0.70, 95% *CI* 0.49 - 0.98, *P =* 0.038); however, in patients aged 35 or older, regardless of whether confounding factors were adjusted for, there was no significant association between high VFA and CPR or LBR (all *P* **>** 0.05). Among patients with a BMI less than 24 kg/m², a high VFA significantly reduced CPR (univariable model: *OR* 0.72, 95% *CI* 0.54 - 0.96, *P =* 0.027; multivariable model: *OR* 0.66, 95% *CI* 0.46 - 0.94, *P =* 0.020) and LBR (univariable model: *OR* 0.73, 95% *CI* 0.54 - 0.98, *P =* 0.034; multivariable model: *OR* 0.65, 95% *CI* 0.45 - 0.92, *P =* 0.015), regardless of confounding factors; in contrast, in patients with a BMI of 24 kg/m² or greater, there was no significant correlation between high VFA and CPR or LBR (*P* **>** 0.05).

**Table 4:** Subgroup analysis for association between high VFA group and clinical outcomes.

Subgroup	Univariable				Multivariable		
	Outcome	*OR* (95%CI)	*P* value	Outcome	*OR* (95%CI)	*P* value	P for interaction
Clinical pregnancy rate							
Age group							0.759
< 35	294/497 (59.2%)	0.83 (0.65 - 1.07)	0.147	294/497 (59.2%)	0.65 (0.46 - 0.93)	0.018	
≥ 35	92/222 (41.4%)	0.80 (0.52 - 1.22)	0.298	92/222 (41.4%)	0.70 (0.39 - 1.28)	0.250	
BMI group							0.363
< 24 kg/m^2^	127/246 (51.6%)	0.72 (0.54 - 0.96)	0.027	127/246 (51.6%)	0.66 (0.46 - 0.94)	0.020	
≥ 24 kg/m^2^	259/473 (54.8%)	0.42 (0.18 - 0.96)	0.04	259/473 (54.8%)	0.54 (0.23 - 1.28)	0.161	
Live birth rate							
Age group							0.901
< 35	250/497 (50.3%)	0.86 (0.67 - 1.09)	0.201	250/497 (50.3%)	0.70 (0.49 - 0.98)	0.038	
≥ 35	72/222 (32.4%)	0.85 (0.54 - 1.34)	0.485	72/222 (32.4%)	0.79 (0.42 - 1.47)	0.451	
BMI group							0.831
< 24 kg/m^2^	105/246 (42.7%)	0.73 (0.54 - 0.98)	0.034	105/246 (42.7%)	0.65 (0.45 - 0.92)	0.015	
≥ 24 kg/m^2^	217/473 (45.9%)	0.70 (0.34 - 1.45)	0.335	217/473 (45.9%)	0.96 (0.44 - 2.08)	0.912	

Low VFA group as reference. OR, odds ratios. Univariable model adjusts for: None. Multivariable model adjusts for: age, BMI, basal E2.

## 4 Discussion

This study, which utilized BIA to measure VFA, provides an in-depth retrospective analysis of data from patients who underwent FET, with the goal of elucidating the complex relationship between visceral fat and pregnancy outcomes. The results revealed a significant correlation between VFA ≥ 65 cm² and diminished ART success, as evidenced by lower CPR and LBR.

Previous research on the impact of excess fat on the pregnancy outcomes of ART has yielded inconsistent results. While some studies have reported no significant correlation between obesity and ART outcomes (18–20), a larger body of clinical research suggests a more nuanced relationship (5, 21–23) which is consistent with our findings. In this study, 4.3% of patients were classified as overweight or obese based on BMI criteria but had a lower VFA, whereas 34.2% were considered normal weight or underweight based on BMI but had a higher VFA. These findings align with Jia et al.’s perspective that BMI alone may no longer be sufficient to accurately assess obesity, and that VFA offers a more precise indication (24, 25). Specifically, in the context of reproductive medicine, measuring VFA provides a more accurate evaluation of a patient’s fertility potential and can help guide more effective and individualized treatment strategies.

In this study, a VFA of ≥65 cm² was associated with poorer pregnancy outcomes following FET. Using this VFA threshold, we observed that patients in the high VFA group were older and had lower basal E2 levels. Previous studies have suggested that advanced age and lower basal E2 levels are linked to adverse reproductive outcomes (26, 27). However, even after adjusting for various confounding factors that could influence pregnancy outcomes, our results still demonstrated a significant negative correlation between VFA and both CPR and LBR, highlighting the potential adverse effects of visceral fat on FET success rates. Excessive fat accumulation may impair endometrial receptivity, with growing evidence indicating that obesity in females can disrupt the conditions necessary for successful implantation. A 2013 study using oocyte donation cycles from healthy-weight donors to isolate the impact of obesity on oocyte and embryo quality found that recipients who were overweight or obese exhibited lower implantation rates, as well as lower CPR and LBR, compared to those with a healthy weight (28). In China, where legislation and regulation regarding oocyte donation remain stringent, many studies have controlled for embryo quality through high-quality autologous embryo transfers, adjusting for potential confounders, and still found that obesity was associated with lower implantation rates, CPR, and LBR (21, 29). These clinical findings are consistent with our conclusions and further refine the understanding of how visceral fat accumulation affects ART pregnancy outcomes. High levels of visceral fat may interfere with healthy embryo implantation and endometrial preparation through several mechanisms, including pro-inflammatory responses (30), hormone regulation (31), and cellular signal disruption (32). A transcriptomic study demonstrated that women with obesity—83.3% of whom had central obesity—showed distinct endometrial gene expression profiles during the embryo implantation window compared to controls (33). This analysis underscores the critical role of visceral fat in reproductive health, suggesting that its impact extends beyond general obesity as indicated by BMI.

The direct association between visceral adiposity and impaired reproductive outcomes highlights the need for targeted interventions to manage and reduce visceral fat in order to improve ART success rates. In our stratified analysis, we found that an increase in VFA levels was significantly correlated with poorer CPR and LBR in women under the age of 35, while no significant correlation was observed in women aged 35 or older. This suggests that the adverse effects of visceral fat accumulation on reproductive health are more pronounced in younger women, whereas in older women, VFA may not be the primary factor influencing reproductive outcomes. Several studies have reported similar findings (5, 6). Among women with a normal or underweight BMI, 43.2% had a high VFA, which was significantly associated with reduced CPR and LBR. This suggests that the specificity of fat distribution, particularly the accumulation of visceral fat, should be an important consideration when assessing fertility in this population. In contrast, no significant correlation was found between VFA and CPR or LBR in the overweight or obese group, likely due to the generally high VFA levels in this cohort—only 4.3% of patients in this group had low VFA, rendering the impact of VFA as a single variable relatively weak in the overall analysis. Therefore, for younger women, even those with a normal or underweight BMI, managing body fat distribution, particularly reducing visceral fat accumulation, should be considered an important strategy for maintaining reproductive health and improving fertility potential. For older women, however, a comprehensive approach that considers multiple factors is necessary to avoid delaying treatment.

In summary, our study utilized BIA to accurately measure VFA, offering a novel approach to examining the effects of visceral fat on ART outcomes. This method provides a more detailed understanding of how specific body composition factors, beyond BMI, influence FET success. Moreover, the adjustment for various confounding factors enhances the robustness and credibility of the findings, facilitating a reliable exploration of the relationship between visceral fat and pregnancy outcomes. However, as this was a single-center retrospective study with a relatively small sample size, the generalizability of the findings to diverse populations with different ethnic backgrounds or health profiles may be limited. Therefore, future prospective studies with larger sample sizes are needed to further investigate the relationship between visceral fat and FET pregnancy outcomes.

## 5 Conclusions

This study identified a significant association between VFA and pregnancy outcomes following FET through an in-depth retrospective analysis. After adjusting for age, BMI, and basal E2 levels, a high VFA was significantly correlated with lower CPR and LBR, with a VFA threshold of 65 cm². This VFA threshold provides a novel benchmark for clinical guidance on adiposity management in ART, potentially enabling more effective and personalized interventions.

## Data Availability

The raw data supporting the conclusions of this article will be made available by the authors, without undue reservation.
