# Supplementary material for: Evaluating the impact of visceral fat on the outcomes of frozen embryo transfer via bioelectrical impedance analysis
Source: Front Endocrinol (Lausanne). 2025 Jan 10;15:1474201. doi: 10.3389/fendo.2024.1474201 (PMC11757113; doi:10.3389/fendo.2024.1474201)
Supplement: Supplementary file 1 [file Table1.docx]

Table S1 Baseline covariates before and after matching.

|  | **Before Matching** | | |  | **After Matching** | | |
| --- | --- | --- | --- | --- | --- | --- | --- |
| **Variables** | **Low VFA Group** | **High VFA Group** | **SMD** |  | **Low VFA Group** | **High VFA Group** | **SMD** |
| No. of patients | 719 | 791 |  |  | 719 | 719 |  |
| Age, years | 31.31 ± 3.95 | 33.28 ± 5.00 | -0.499 |  | 31.31 ± 3.95 | 32.39 ± 4.25 | -0.273 |
| Endometrial preparation protocols, n (%) |  |  |  |  |  |  |  |
| NC | 187/719 (26.0%) | 158/719 (20.0%) | 0.138 |  | 187/719 (26.0%) | 153/719 (21.3%) | 0.108 |
| OI | 50/719 (7.0%) | 71/719 (9.0%) | -0.079 |  | 50/719 (7.0%) | 63/719 (8.8%) | -0.071 |
| HRT | 482/719 (67.0%) | 562/719 (71.0%) | -0.085 |  | 482/719 (67.0%) | 503/719 (70.0%) | -0.062 |
| Transfer embryo type, n (%) |  |  |  |  |  |  |  |
| D3 | 144/719 (20.0%) | 197/719 (24.9%) | -0.122 |  | 144/719 (20.0%) | 161/719 (22.4%) | -0.059 |
| D5/D6 | 575/719 (80.0%) | 594/719 (75.1%) | 0.122 |  | 575/719 (80.0%) | 558/719 (77.6%) | 0.059 |

*Abbreviations*: SMD, Standardized Mean Difference; NC, natural cycle; OI, ovulation induction; HRT, hormone replacement therapy.
